# Interesting to Dentists

**Published:** 1868-02

**Authors:** 


### EDITORIAL DEPARTMENT.

*Interesting to Dentists.* In the December number of the *Dental Register* we find the following which will prove interesting to our readers:

"*Legal Contest with the Dentists.*—A motion was made in the United States District Court in the cases of Goodyear *et al.* vs. Taft, Berry, Smith, and seven others, for a preliminary injunction, based upon the late decision of Judge Nelson in the Vulcanite case. The defendants replied that they had abandoned the Goodyear compound, and were now using a rubber prepared by the Porter Manufacturing Company, under the patent granted to Edward L. Simpson, October 16, 1866. Affidavits were filed to prove that this compound contained only two and a half ounces of sulphur to a pound of rubber; the minimum fixed by the Goodyear patent being four ounces. Upon the day appointed for a hearing, both parties appearing, the complainant's counsel withdrew his motion, and abandoned the application for an injunction in all the cases."

Drs. A. Berry and H. R. Smith speak very favorably of Hale's rubber under the Simpson patent. Dr. Berry says he has used all the different preparations of dental rubber, and that after six weeks exclusive use of the "Improved Dental Rubber" manufactured by A. R. Hale, under Simpson's patent, issued Oct. 16th, 1866, he prefers it to any he has seen.

He also remarks that "the Simpson rubber requires more heat or longer time than the Goodyear for vulcanizing. If the "Directions for Steaming" accompanying the Simpson gum are followed, it is of a lighter shade of color than if steamed a shorter time; but as I regard this of little importance, I run up the mercury to 330 or 335, and steam from fifty to fifty-five minutes. Of course the necessary time may differ in different vulcanizers according as they differ in preventing escape of steam."

Dr. Smith speaks of this rubber as follows:—"Feeling it a duty for all members of the profession to call attention to improvements in Dental work, I would say that I have been using the rubber made by A. R. Hale, under the Simpson patent, and find it equal to the best and superior to most of the rubber used for Dental purposes; and would advise all Dentists to give it a trial, and think they will be highly pleased with it."

